# Editorial: The hormonal control of shoot organogenesis and morphogenesis: from leaves to seeds

**DOI:** 10.3389/fpls.2023.1319859

**Published:** 2023-11-07

**Authors:** Hélène S. Robert, Marcus G. Heisler, Langtao Xiao, Benoit Landrein

**Affiliations:** ^1^Hormonal Crosstalk in Plant Development, Mendel Center for Plant Genomics and Proteomics, Central European Institute of Technology, Masaryk University (CEITEC MU), Brno, Czechia; ^2^School of Life and Environmental Sciences, University of Sydney, Sydney, NSW, Australia; ^3^College of Bioscience and Biotechnology, Hunan Agricultural University, Changsha, China; ^4^Laboratoire Reproduction et Développement des Plantes, Université de Lyon, ENS de Lyon, UCB Lyon, CNRS, INRAE, INRIA, Lyon, France

**Keywords:** hormones, plant hormones, organogenesis, shoot development, morphogenesis, plant architecture

## Introduction

Phytohormones shape the plant above and below ground to enable grounding, interaction with the soil microbiome and with surrounding plants, water and nutrient uptake, organ production, photosynthesis optimization, and seed production. Plant aerial architecture results from the dynamic processes of shoot branching, leaf and flower production following phyllotaxis, and reproduction resulting from the post-embryonic activity of the shoot apical meristem. Hormones influence and coordinate plant development and physiology at the organismal level. Different hormones regulate different aspects of shoot development, acting together or antagonistically. This Research Topic consists of seven articles, e.g., four original research articles and three reviews, dealing with the influence of hormones on shoot development, branching regulation, flower aging, and fruit maturation. They show that all these processes are influenced by many different hormones, such as ethylene, gibberellins, abscisic acid, auxin, cytokinin, and strigolactones.

## Shoot organogenesis and branching processes - a hub for hormonal crosstalk

Post-embryonic shoot organogenesis depends on the activity of the shoot apical meristem (SAM). The SAM contains a pool of slow-growing stem cells in the central zone (CZ), surrounded by fast-growing cells in the peripheral zone (PZ), where organs are initiated. It is structured in three cell layers. The SAM is maintained by the WUSCHEL (WUS) – CLAVATA 3 (CLV3) module (Greb and Lohmann, 2016). Cytokinin production occurs in the SAM, and cytokinin signaling regulates WUS expression in the OC, making cytokinins a key player in SAM maintenance. Cytokinins work closely with auxin to determine the position and timing of the leaf and floral primordia initiation at the flanks of the SAM, which is instrumental for phyllotaxis (Shi and Vernoux, 2022). In addition to their role in SAM maintenance, cytokinins have also been identified as critical molecules for the induction of *de novo* shoot organogenesis (Melnyk, 2023). The developmental plasticity of plants enables the *in vitro* regeneration of a whole plant from explants. Hormone combinations promote tissue regeneration and the formation of new organs (root and shoot) from callus tissue. The review by Šmeringai et al. discusses the function of cytokinins during *in vitro* shoot regeneration in Arabidopsis and details the molecular regulators involved in this process. Optimizing the regeneration of transformed explants is the bottleneck of any protocol for generating transgenic crops that cannot be transformed by floral dipping. In the 1950s, Skoog and Miller found that combinations of auxin and cytokinin treatments could be used to induce explant rooting and shooting (Melnyk, 2023). However, specific optimization of the hormonal composition of the shoot-inducing medium is often necessary to enhance shooting in recalcitrant species. Accordingly, George et al. found that thiophene acetic acid (TAA), a novel auxin analog derived from the degradation of ticarcillin, an antibiotic used against *Agrobacterium*, enhances root and shoot regeneration in tomato explants. These effects of TAA on tissue regeneration appear to be similar to those of other natural and synthetic auxins.

Branching is another process that modulates shoot architecture. In a Perspective article, Kebrom and Doust discussed the process of axillary bud outgrowth in grasses. Axillary buds are formed from a few meristematic cells at the axil of a leaf, regardless of growth conditions. However, the decision for the bud to grow into a branch or to become dormant depends on endogenous and environmental cues. The authors reviewed the impact of a shift from symplastic to apoplastic sugar transport in the bud on its outgrowth. In annual plants, this shift would be regulated by different plant hormones (cytokinins, abscisic acid, auxin, and strigolactones) and light quality. Light is key for sugar production through photosynthesis. Luo et al. identified auxin as a repressor of chlorophyll synthesis, which affects photosynthetic efficiency. In a Research article, Liu et al. investigated stem outgrowth in cherry rootstocks. Stem development determines the shape of the cherry trees. The authors studied how strigolactones modulate stem outgrowth in cherry rootstocks, with the potential to produce dwarf cherry trees for high-density cultivation.

## Flower aging and fruit maturation - which essential hormones regulate senescence signaling?

Senescence, fruit maturation, and ripening are natural events that occur during the aging process of leaves, flowers, and fruits. Leaf senescence alters leaf productivity by reducing photosynthetic performance and remobilizing water and nutrients. Senescence is a tightly regulated and coordinated process in which cellular changes lead to visible phenotypes such as color changes, wilting, or fading. It is influenced by abiotic stresses (Sade et al., 2017). Therefore, it is associated with different hormones, ethylene, cytokinin, gibberellins, and abscisic acid, through gene regulatory networks involving key transcriptional factors. By controlling leaf senescence and (in)directly impacting plant morphogenesis, hormones promote the remobilization of N resources from leaves to contribute to seed and fruit development.

Ji et al. investigated senescence in cut flowers of the herbaceous peony. Petal senescence in peonies is associated with decreased levels of gibberellins and increased levels of ethylene and abscisic acid. The authors identified a MYB transcription factor, PlMYB308, and showed that it is upregulated in senescent petals, induced by abscisic acid, and repressed by gibberellins. Genetic manipulation of *PlMYB308* expression levels appears to link the transcription factor to the regulation of flower senescence through modulation of hormone levels, as PlMYB308 regulates the expression of several ethylene-producing enzymes.

A related process is fruit ripening, during which the fruit is modified by changing color, improving flavor, and softening its flesh. This developmental process involves multiple hormones and transcriptional regulatory networks that control the expression of ripening-related genes. Fleshy fruits are categorized as climacteric and non-climacteric based on the type of hormonal regulation of the ripening processes (Kou et al., 2021). The beginning of the ripening process in climacteric fruits (such as apples, bananas, and tomatoes) depends on a burst of ethylene (Brumos, 2021). It can be repressed by inhibiting ethylene production or activity. This has been exploited to control the storage and shelf life of these fruits by decreasing atmospheric oxygen levels. Ripening in non-climacteric fruits depends on abscisic acid and other hormones, as discussed in a mini-review article by Fan et al.. The type of hormonal regulation depends on the type of non-climacteric fruit (e.g., the structure and tissue origin of the fruit). Strawberry is often used as a model non-climacteric fruit. It has been demonstrated that ripening in strawberries depends on the hormonal communication between achenes and receptacles. But how does this work in non-climacteric fruits that do not possess achenes with different textures and anatomical features? The authors discussed the issue of finding a good research model to study ripening and its hormonal regulation in non-climacteric fruits.

In summary, this Research Topic brings together recent findings and literature that demonstrate the complexity and diversity of hormonal regulation of plant development above ground.

## Author contributions

HR: Conceptualization, Writing – original draft, Writing – review & editing. LX: Writing – review & editing. BL: Writing – review & editing. MH: Writing – review & editing.
